# Changes in Treg and Breg cells in a healthy pediatric population

**DOI:** 10.3389/fimmu.2023.1283981

**Published:** 2023-11-21

**Authors:** Yiyi Luo, Daniel Acevedo, Alexandru Vlagea, Anna Codina, Ana García-García, Angela Deyà-Martínez, Celia Martí-Castellote, Ana Esteve-Solé, Laia Alsina

**Affiliations:** ^1^ Clinical Immunology and Primary Immunodeficiencies Unit, Allergy and Clinical Immunology Department, Hospital Sant Joan de Déu, Barcelona, Spain; ^2^ Clinical Immunology Unit, Hospital Sant Joan de Déu-Hospital Clínic, Barcelona, Spain; ^3^ Study Group for Immune Dysfunction Diseases in Children (GEMDIP), Institut de Recerca Sant Joan de Déu, Barcelona, Spain; ^4^ Biomedic Diagnostic Center (CDB), Hospital Clínic of Barcelona, Clinical Immunology Unit Hospital Sant Joan de Déu-Hospital Clínic de Barcelona, Barcelona, Spain; ^5^ Biobanco Pediátrico para la Investigación Hospital Sant Joan de Déu, Barcelona, Spain; ^6^ Department of Surgery and Medical Specializations, Facultat de Medicina i Ciències de la Salut, Universitat de Barcelona, Barcelona, Spain

**Keywords:** Treg cell, Breg cell, FoxP3, immunophenotyping, IPEX, child

## Abstract

The interpretation of clinical diagnostic results in suspected inborn errors of immunity, including Tregopathies, is hampered by the lack of age-stratified reference values for regulatory T cells (Treg) in the pediatric population and a consensus on which Treg immunophenotype to use. Regulatory B cells (Breg) are an important component of the regulatory system that have been poorly studied in the pediatric population. We analyzed (1) the correlation between the three immunophenotypic definitions of Treg (CD4^+^CD25^hi^CD127^low^, CD4^+^CD25^hi^CD127^low^FoxP3^+^, CD4^+^CD25^hi^FoxP3^+^), and with CD4^+^CD25^hi^ and (2) the changes in Treg and Breg frequencies and their maturation status with age. We performed peripheral blood immunophenotyping of Treg and Breg (CD19^+^CD24^hi^CD38^hi^) by flow cytometry in 55 healthy pediatric controls. We observed that Treg numbers varied depending on the definition used, and the frequency ranged between 3.3–9.7% for CD4^+^CD25^hi^CD127^low^, 0.07-1.6% for CD4^+^CD25^hi^CD127^low^FoxP3^+^, and 0.24-2.83% for CD4^+^CD25^hi^FoxP3^+^. The correlation between the three definitions of Treg was positive for most age ranges, especially between the two intracellular panels and with CD4^+^CD25^hi^ vs CD4^+^CD25^hi^CD127^low^. Treg and Breg frequencies tended to decline after 7 and 3 years onwards, respectively. Treg’s maturation status increased with age, with a decline of naïve Treg and an increase in memory/effector Treg from age 7 onwards. Memory Breg increased progressively from age 3 onwards. In conclusion, the number of Treg frequencies spans a wide range depending on the immunophenotypic definition used despite a good level of correlation exists between them. The decline in numbers and maturation process with age occurs earlier in Breg than in Treg.

## 1 Introduction

Regulatory T cells (Treg) are a main component of immune regulation and tolerance; quantitative (frequency) and/or qualitative (function) defects in Treg lead to autoimmunity, inflammation, lymphoproliferation, and/or severe atopy (1–3). Tregopathies are a growing group of primary immune regulatory disorders (PIRD) (4, 5) in which Treg development and function are directly affected. At present, mutations in 10 genes are responsible for Treg defects, and these are either loss-of-function mutations [forkhead box P3 (*FOXP3*), cluster of differentiation 25 (*CD25)* (interleukin 2 receptor A), *CD122* (interleukin 2 receptor B), cytotoxic T-lymphocyte-associated protein 4 (*CTLA-4*), LPS Responsive Beige-Like Anchor Protein (*LRBA*), broadcomplex-tramtrack-bric-a-brac and Cap’n’collar homology 2 (*BACH2*), FERM domain containing kindlin 1 (*FERMT1*), and DEF6 guanine nucleotide exchange factor (*DEF6*)] or gain-of-function mutations [signal transducer and activator of transcription 3 (*STAT3*) and IKAROS family zinc finger 1 (*IKZF1*)] (2, 3). Apart from the aforementioned genes, recent findings reported that the Neurobeachin Like 2 (*NBEAL2*) interacts with CTLA-4 and thereby up-regulates CTLA-4 expression signaling (6). As might be expected, the loss of function mutation in *NBEAL2* leads to a secondary CTLA-4 deficiency in activated T cells; however, Treg function appears to be unaffected (6). Therefore, further studies are needed to evaluate the impact of NBEAL2 deficiency on Treg phenotype and activity.

The diagnostic approach for suspected inborn errors of immunity (IEI) including Tregopathies consists of what is defined as the “wholly trinity approach” which includes medical history, genetic studies, and immunological tests (2, 3, 7). Next-generation sequencing (NGS) has made genetic diagnosis more affordable and approachable and it is a powerful tool for targeted therapy (8). Nonetheless, genetic studies present limitations including a lower than desirable diagnostic yield, which is below 30-40% in pediatric patients (8), and the difficulty in interpreting the causal relationship between genotype and clinical phenotype (8–11). New multidisciplinary models are being implemented to resolve these limitations (12). In fact, the challenges are greater for variants of unknown significance and also for phenotypes without confirmatory genetics as locus heterogeneity and incomplete penetrance make it difficult to draw firm conclusions in cause-effect relationship (8, 9, 13–16). Current guidelines for investigating the causality of sequence variants in IEI incorporate immunological tests including immunophenotyping mostly by flow cytometry to assess the biological effects of mutated genes (9, 10, 13, 16–20). Immunophenotyping is also included in different international consensus documents to enable the clinical diagnosis of IEI in the absence of a genetic diagnosis (13, 21, 22).

The first described and best-known Treg defect is the immune dysregulation polyendocrinopathy enteropathy X-linked syndrome (IPEX). IPEX is caused by hemizygous pathogenic variants in the *FOXP3* gene (15). There are over 70 *FOXP3* mutations associated with IPEX (15, 17, 18, 23) but still no well-defined genotype-phenotype correlation which can interfere with patient diagnosis and decisions on therapeutic interventions (24). Currently, 20-30% of patients presenting clinical features of IPEX have no mutations in *FOXP3* and are termed “IPEX-like” (25). In the European Society for Immunodeficiencies (ESID) Registry working definitions for the clinical diagnosis of IEI (13, 21), evaluation of Treg (Foxp3 expression in CD4^+^CD25^+^ cells) appears as a diagnostic criterion in IPEX and IPEX-like suspected disease (13). Furthermore, evaluation of Treg may also be of value in other IEI besides Tregopathies, such as hemophagocytic lymphohistiocytosis (HLH) (26), very early onset inflammatory bowel disease (VEO-IBD) (27), autoimmune lymphoproliferative syndrome (ALPS) (28, 29), activated phosphoinositide 3-kinase delta syndrome (APDS) (28, 30), and common variable immunodeficiency (CVID) (31–33).

Tregopathies, and most PIRD, are early-onset diseases (18, 19, 34). Thus, Treg assessment in the healthy pediatric population is needed for the interpretation of patients’ results. This has only been previously performed in two smaller cohorts (35, 36). Furthermore, it can help to better characterize Treg norm-biological development during early life as it shapes the future regulatory immune system. An outstanding question is the markers used to define Treg: the most accurate definition for clinical diagnosis includes the use of both surface and intracellular markers: CD4^+^CD25^hi^CD127^low^FoxP3^+^ (25, 37–39). However, current literature reports three other different marker combinations for both research and clinical use: CD4^+^CD25^hi^CD127^low^ cells (35, 40), CD4^+^CD25^hi^FoxP3^+^ cells (18, 41, 42), and CD4^+^CD25^hi^CD127^low^CCR4^+^ (43, 44). As for the latter, C-C chemokine receptor 4^+^ (CCR4^+^) is highly expressed in Treg and plays a key role in Treg infiltration to the inflammatory tissue (45), thus CCR4 should be used as an additional marker applied for defining memory Treg (CD45RO^+^) with effector capacity called effector Treg (eTreg) (36, 37, 46). Therefore, herein, we defined CD4^+^CD25^hi^CD127^low^CD45RO^-^CCR4^-^ as naïve Treg and CD4^+^CD25^hi^CD127^low^CD45RO^+^CCR4^+^ as eTreg. Finally, the expression of IL-2R (CD4^+^CD25^hi^) is of clinical use in Treg evaluation as well (13, 21). This lack of agreement on the definition of the Treg phenotype marker highlights the need to evaluate whether a variation exists in Treg numbers depending on the marker combinations used in the absence of a consensus, especially when intended for clinical use (39).

Regulatory B cells (Breg) or interleukin (IL)-10 producing B cells (B10) are also widely accepted as an important modulatory component of the immune system that suppresses T cell differentiation and promotes peripheral tolerance (47–49). Specifically, Breg suppresses T helper (Th) 1/17 cells differentiation and their capacity to release inflammatory cytokines (i.e., IFN-γ and TNF-α) (48, 50); Breg also enhances the activity of Treg (48). Thus, the breakdown of Breg activity is assumed to be associated with both autoimmunity (49, 51, 52) and immunodeficiency (53–55). In contrast to Treg, one of the main challenges in defining Breg is the lack of consensus on their phenotypic definition and the identification of the lineage-specific transcription factor (47). Currently, the most widely accepted phenotype for Breg is CD19^+^CD24^hi^CD38^hi^ (immature cells) (48, 50, 56–59), and for memory Breg, it is CD19^+^CD24^hi^CD27^+^ (also IL-10 producing cells) (60, 61). However, this definition of Breg remains controversial. Expression of both CD24 and CD38 is highly present in bone marrow-derived immature B cells, so many authors have described CD19^+^CD24^hi^CD38^hi^ cells as transitional B cells (36, 62). Nevertheless, several works have demonstrated that these CD19^+^CD24^hi^CD38^hi^ cells do exhibit inhibitory capacity (47, 48, 62, 63), whereas CD19^+^CD24^int^CD38^int^ cells do not (50), which further reinforces the notion that CD19^+^CD24^hi^CD38^hi^ are B cells with regulatory functions. Unlike Treg, Breg is only rarely evaluated in the context of IEI (33). Nonetheless, it is of interest to define Breg’s norm-biological development during early life for future work (53–55).

In summary, our aim was to study the correlation between the three currently most used Treg definitions, and with CD4^+^CD25^hi^, to find out whether they are comparable, and then to evaluate the changes in both Treg (three definitions) and Breg in the pediatric population, which could help improve the understanding of the development of the regulatory population’s biological process in health and disease, and their use in the clinical diagnosis of IEI.

## 2 Methods

### 2.1 Sample collection

Peripheral blood was collected in vacutainer tubes containing lithium heparin as an anticoagulant (Becton Dickinson, Cat 367885, Franklin Lakes, New Jersey, United States) and maintained at room temperature until processing within 24h after collection.

The control population of pediatric patients was recruited with the support of the Hospital Sant Joan de Déu-Biobank, following the circuit established by the Biobank for this objective and after signing the specific informed consent. Included healthy pediatric controls were patients receiving elective surgery (i.e., ear, nose, and throat surgery; phimosis surgery) supervised by pediatricians from the Hospital Sant Joan de Déu. *Inclusion criteria*: a) age under 18 years of age; b) signing of informed consent and assent specific to the healthy controls. *Exclusion criteria*: a) diagnosis of chromosomal diseases, cardiac or midline malformations, and oncological, hematological, or immune-related diseases; b) presenting any type of acute or chronic infection known at the time of blood sample collection.

### 2.2 Sample processing

For surface staining of both Treg and Breg, 50µL of heparinized whole blood was incubated for 15 min at room temperature (RT) with the appropriate concentration of mAbs. Cells were then incubated with 2 ml of BD lysing solution 1x (BD Bioscience, United States) for 15 min at RT to lyse erythrocytes and fix cells. Finally, cells were washed two times with phosphate-buffered saline (PBS) 1X. For Treg intracellular staining, Treg Detection Kit (CD4/CD25/FoxP3) (Cat: 130-093-142, Miltenyi Biotec, Bergisch Gladbach, Germany) was used following the manufacturer’s instructions. Briefly, after surface mAb staining, cells were fixed with 500μl of fixation buffer for 30 min at 4°C. Cells were washed two times with PBS 1X and after that incubated with perm buffer for cell permeabilization. To block non-specific mAb binding, cells were incubated with 20μl of perm buffer and 5μl of FcR Blocking Reagent (Cat: 130-059-901, Miltenyi Biotec, Bergisch Gladbach, Germany) for 5 min at RT. Cells were then stained with FoxP3-APC mAb for 30 min at 4°C and finally washed with PBS 1X.

Populations were defined as follows: 1) Treg as CD4^+^CD25^hi^CD127^low^, CD4^+^CD25^hi^CD127^low^FoxP3^+^, and CD4^+^CD25^hi^FoxP3^+^, and 2) Treg subsets as naïve Treg (CD4^+^CD25^hi^CD127^low^CD45RO^-^CCR4^-^), eTreg (CD4^+^CD25^hi^CD127^low^CD45RO^+^CCR4^+^), and activated eTreg (CD4^+^CD25^hi^CD127^low^CD45RO^+^CCR4^+^HLA-DR^+^) (**Supplementary Figure 1**). We studied Treg with (1) an extracellular panel including CD3-APC-H7 (Cat: 641415), CD4-V450 (Cat: 651849), CD25-PE (Cat: 555432), CD127-PE-Cy7 (Cat: 560822), CD45RO-APC (Cat: 340438), C-C chemokine receptor type 4 (CCR4)-BV510 (Cat: 563066), and human leukocyte antigens-DR (HLA-DR)-FITC (Cat: 555811) and (2) an intracellular panel including CD3-V450 (Cat: 560365), CD4-FITC (Cat: 345768), CD25-PE (Cat: 555432), CD127-PE-Cy7 (Cat: 560822), and FoxP3-APC (130-125-580). Transitional Breg was defined as CD19^+^CD24^hi^CD38^hi^ and memory Breg as CD19^+^CD24^hi^CD27^+^. We studied Breg with CD19-BV510 (Cat: 562947), CD24-PerCP-Cy5.5 (Cat: 561647), CD38-PE-Cy7 (Cat: 335825), and CD27-APC (Cat: 558664). Monoclonal antibodies (mAbs) used for all panels were from BD Biosciences (Franklin Lakes, New Jersey, United States), with the exception of anti-FoxP3 mAb which was from Miltenyi Biotec, Bergisch Gladbach, Germany.

### 2.3 Sample acquisition and statistical analysis

All samples studied with flow cytometry were acquired using a FACSCanto-II (BD Bioscience) cytometer. A minimum of 20,000 events were acquired for the different populations studied: T cells for Treg and B cells for Breg flow cytometry data were analyzed with Flowjo v.10.

We used SPSS 19.0 (AN BIM^®^ Company) for the statistical analysis. The normal range of each cell subset was defined in both absolute count (cells 10^9^/L) and relative frequency (% populations) based on the median, minimum, and maximum. The absolute number of subsets was calculated from the absolute number of lymphocytes provided by the hematological analyzer (ADVIA 2120, Siemens, Germany). As data did not follow a Gaussian distribution, we performed non-parametric tests to study the significance of the correlation between cell subset/age (Spearman test) and the comparisons between the age groups (Mann-Whitney U test). In the Spearman test, the perfect negative correlation was referred to as -1 and the perfect positive correlation as +1. Low positive association was 0.1-0.3; moderate positive association was between 0.3-0.5; and strong positive association was 0.5-1 (64). Negative correlations follow the same criteria. We used Prism 7.04 software (GraphPad, La Jolla, CA, USA) for the graphical representation.

This study was carried out in accordance with the recommendations of the Ley General de Sanidad (25/4/1986) Art. 10. The protocol was approved by the Ethics Committee of the Hospital Sant Joan de Déu (Comité Ético de Investigaciones Clínicas number PIC-129-18). All parents/legal guardians of children included in this study signed the informed consent, complying with current legislation.

## 3 Results

The study cohort included 55 peripheral blood samples from healthy pediatric donors aged 1 to 18 years old: 12 aged 1 - 3 years (11 boys, 1 girl); 6 aged >3 - 5 years (6 boys); 9 aged >5 - 7 years (7 boys, 2 girls); 14 aged >7 - 10 years (11 boys, 3 girls); 6 aged >10 - 14 years (2 boys, 4 girls); and 8 aged >14 - 18 years (4 boys, 4 girls), all Caucasian.

### 3.1 Correlation between the different definitions of Treg and CD4^+^CD25^hi^

**Figure 1:**
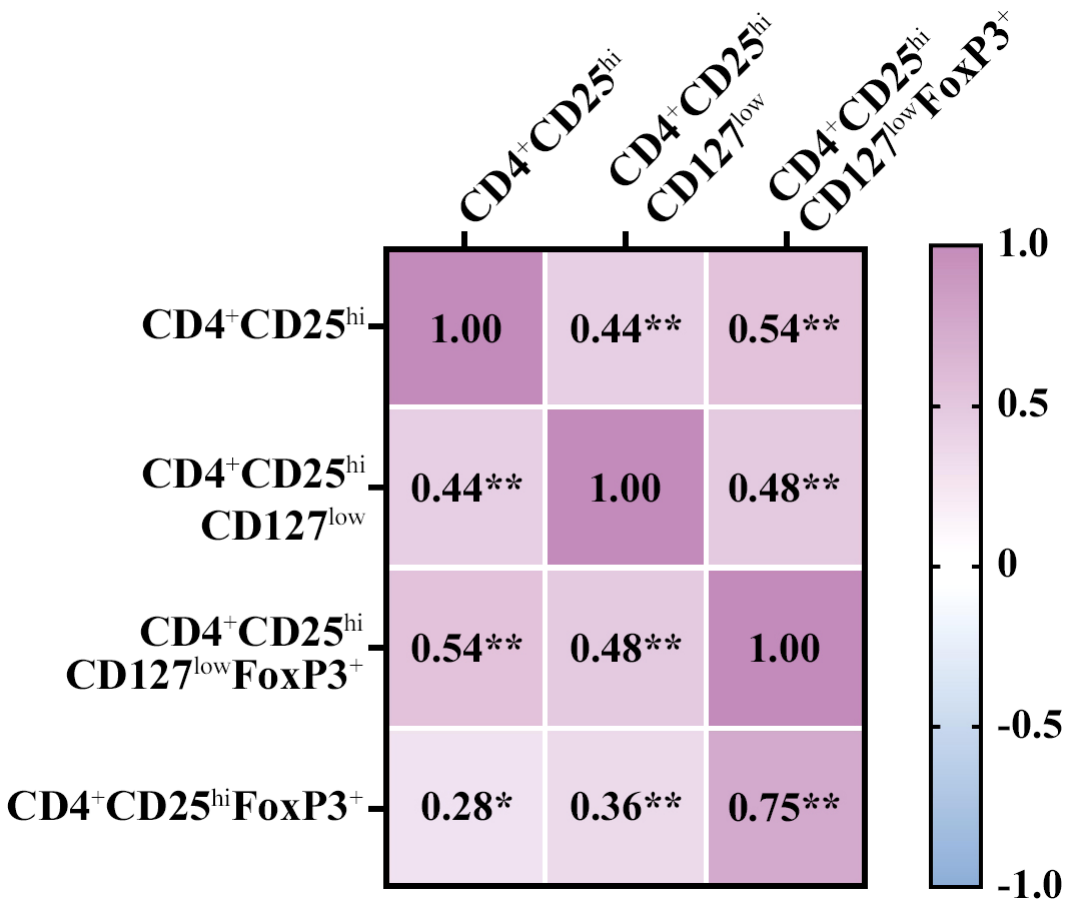
Correlation between gating strategies of regulatory T cell (Treg) and CD4^+^CD25^h^ **^i^.** The three Treg definitions are CD4^+^CD25^hi^CD127^low,^CD4^+^CD25^hi^CD127^low^Foxp3^+^, and CD4^+^CD25^hi^FoxP3^+^. The overall correlation was positive moderate-strong between the Treg definitions, with the similarity between the intracellular panels being especially remarkable. The values inside the boxes indicate the Spearman correlation strength. Low association was between 0.1-0.3; moderate positive association was between 0.3-0.5; and strong positive association was 0.5-1. (*) p value ≤ 0.05; (**) p value ≤ 0.01.

Firstly, we tested the comparability of the definitions of Treg including both extracellular (CD4^+^CD25^hi^CD127^low^) and intracellular panels (CD4^+^CD25^hi^CD127^low^FoxP3^+^ and CD4^+^CD25^hi^FoxP3^+^). Overall, we observed: 1) a moderate positive correlation (r = 0.3-0.5) between the extracellular panel and the two intracellular panels and (2) a strong positive correlation between the two intracellular panels (r = 0.750; p = 9.21E-15); all results mentioned were statistically significant (**Figure 1**). Then, we analyzed the correlations by age range (**Supplementary Figure 3**) and the results suggested a strong correlation between the two intracellular panels for most age ranges. Concretely, the correlation between CD4^+^CD25^hi^FoxP3^+^ and CD4^+^CD25^hi^CD127^low^FoxP3^+^ for the age groups 1-3 years (r = 0.678; p = 0.015), 7-10 years (r = 0.713; p < 0.009), and 14-18 years (r = 0.943; p < 0.005) was strongly positive.

In addition, due to the importance of CD4^+^CD25^hi^ for the diagnosis of PIRD (21), we analyzed the correlation between CD4^+^CD25^hi^ and the three Treg definitions. Overall, we saw a low positive correlation (r = 0.28) with CD25^hi^FoxP3^+^, a moderate positive correlation (r = 0.44) with CD4^+^CD25^hi^CD127^low^, and a strong positive correlation (r = 0.54) with CD4^+^CD25^hi^CD127^low^FoxP3^+^; the results mentioned were statistically significant (**Figure 1**). However, when analyzing the comparison by age range, the definitions CD4^+^CD25^hi^ and CD4^+^CD25^hi^CD127^low^ were comparable for most age groups with a moderate-strong correlation: 1-3 years (r = 0.755; p = 0.005), 5-7 years (r = 0.9; p < 0.037), and 7-10 years (r = 0.771; p < 0.003) (**Supplementary Figure 3**). In summary, the frequency of CD4^+^CD25^hi^ showed a positive correlation with both CD4^+^CD25^hi^CD127^low^ and CD4^+^CD25^hi^CD127^low^FoxP3^+^, and this correlation was more prominent with CD4^+^CD25^hi^CD127^low^ by age range.

### 3.2 Marked variability in total Treg frequency with the different gating definitions and with age

**Figure 2:**
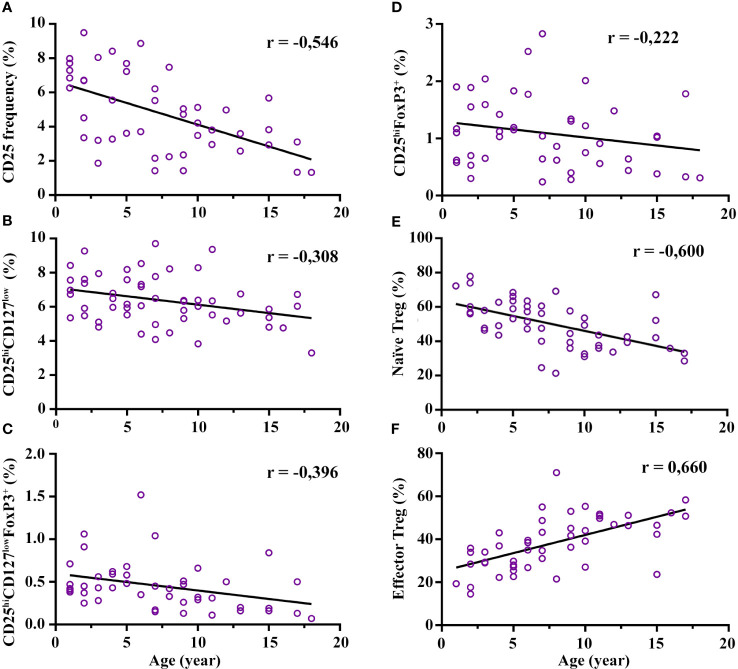
Correlation of regulatory T cell (Treg) frequency with age. Total Treg frequency decreases with age along with a maturation process. Specifically, **(A–D)** represent a negative correlation of CD25 expression and total Treg frequency with age (three definitions), each with its degree of Spearman correlation strength. Graph **(E)** represents a strong negative correlation of naïve Treg (CD4^+^CD25^hi^CD127^low^CD45RO^-^CCR4^-^) with age and graph **(F)** represents a strong positive correlation of effector Treg (CD4^+^CD25^hi^CD127^low^CD45RO^+^CCR4^+^) with age. All correlations reached statistical significance. The frequency of CD25, CD25^hi^CD127^low^, CD25^hi^CD127^low^FoxP3^+^, and CD25^hi^FoxP3^+^ are calculated from CD4^+^ cells. The naïve and effector Treg cells are calculated from CD25^hi^CD127^low^ cells. Low association was between 0.1-0.3; moderate positive association was between 0.3-0.5; and strong positive association was between 0.5-1.

The Treg subset relative frequency (%) and absolute counts are shown in **Table 1A**. In broad terms, we observed large differences in the relative frequency range of Treg depending on the gating strategy (from 1-18 years): 3.3–9.7% for CD4^+^CD25^hi^CD127^low^, 0.07-1.6% for CD4^+^CD25^hi^CD127^low^FoxP3^+^, and 0.24-2.83% for CD4^+^CD25^hi^FoxP3^+^; the % is from CD4^+^ cells. The CD4^+^CD25^hi^ frequency presented a strong decline with age (r = -0.546; p = 1.05E-4), mainly from the age of 7 years onwards (**Figure 2A**; **Supplementary Figure 4A**). This decline was consistent with the decrease in the total Treg frequency: there was a moderate negative correlation with age regarding the gating definitions CD4^+^CD25^hi^CD127^low^ (r = -0.308; p = 0.024) and CD4^+^CD25^hi^CD127^low^FoxP3^+^ (r = -0.396; p = 0.007), respectively (**Figures 2B, C**). The decline of CD4^+^CD25^hi^CD127^low^ FoxP3^+^ was particularly notable from the age of 7 years onwards with a median drop from 0.68% at 5-7 years to 0.325% at 7-10 years (p = 0.015) (**Figure 2C**; **Supplementary Figure 4C**). Interestingly, we observed a tendency for CD4^+^CD25^hi^FoxP3^+^ to increase from 3 to 7 years compared to the youngest (1-3 years) (median 0.9% at 1-3 years vs 1.77% at 5-7 years; p = 0.045), although it did not reach statistical significance at 3-5 years. After 7 years of age, the CD4^+^CD25^hi^FoxP3^+^ frequency decreases by half until 18 years (**Figure 2D**; **Supplementary Figure 4D**). In summary, the frequency of Treg showed a broad variability depending on the immunophenotypic definition used, and a marked reduction with age, mainly after the age of 7 years.

Table 1Age-stratified values of regulatory T and B cells (Treg/Breg) in both relative frequency (%) and absolute count (10^9^/L).

**Table:** 

A) Treg and subset values.														
Treg/subset	From	Cell frequency	1-3 years		> 3-5 years		> 5-7 years		> 7-10 years		> 10-14 years		>14-18 years	
			(n)	Median (min-max)	(n)	Median (min-max)	(n)	Median (min-max)	(n)	Median (min-max)	(n)	Median (min-max)	(n)	Median (min-max)
CD3+CD4+CD25hi (IL-2 receptor)	CD3+ CD4+	%	12	**6.8** (1.9-9.5)	5	**5.55** (3.2-8.41)	5	**6.21** (3.61-8.86)	12	**3.85** (1.42-7.47)	5	**3.59** (2.57-4.97)	6	**3.02** (1.32-5.67)
		10^9^cells/L	12	**0.1** (0.01-0.17)	5	**0.03** (0.006-0.1)	5	**0.03** (0.006-0.083)	12	**0.015** (0.002-0.08)	5	**0.01** (0.003-0.02)	6	**0.07** (0.001-0.03)
CD3+CD4+CD25hiCD127low (Treg: extracellular panel)	CD3+CD4+	%	12	**7.2** (4.8-9.3)	6	**6.06** (5.1-6.79)	9	**7.31** (4.4-8.53)	14	**6.17** (3.83-9.7)	6	**5.99** (5.16-9.36)	7	**5.37** (3.29-6.72)
		10^9^cells/L	12	**0.1** (0.025-0.17)	6	**0.03** (0.01-0.06)	9	**0.03** (0.01-0.08)	14	**0.02** (0.004-0.1)	6	**0.015** (0.01-0.04)	7	**0.012** (0.002-0.03)
CD3+CD4+CD25hiCD127low Foxp3+ (Treg: intracellular panel)	CD3+CD4+	%	12	**0.4** (0.25-1.06)	5	**0.58** (0.43-0.62)	5	**0.68** (0.35-1.52)	12	**0.33** (0.13-0.66)	5	**0.2** (0.11-0.5)	6	**0.18** (0.07-0.84)
		10^9^cells/L	12	**0.004** (0.001-0.02)	5	**0.003** (0.001-0.005)	5	**0.003** (0.001-0.014)	12	**0.001** (0.0001-0.01)	5	**0.001** (0.0001-0.002)	6	**0.0004** (0.00004-0.004)
CD3+CD4+CD25hiFoxp3+ (Treg: intracellular panel)	CD3+CD4+	%	12	**0.9** (0.3-1.9)	5	**1.42** (1.03-2.04)	5	**1.77** (1.15-2.83)	12	**0.81** (0.24-2.01)	5	**0.64** (0.44-1.48)	6	**0.7** (0.31-1.78)
		10^9^cells/L	12	**0.008** (0.002-0.034)	5	**0.007** (0.002-0.02)	5	**0.01** (0.002-0.03)	12	**0.003** (0.0002-0.02)	5	**0.002** (0.001-0.01)	6	**0.002** (0.0002-0.01)
CD3+CD4+CD25hi CD127lowCCR4-CD45RO- (Naïve Treg)	CD4+ CD25hi CD127low	%	8	**59** (46.4-77.9)	6	**51.05** (43.5-66.1)	9	**58.9** (47.2-68.4)	14	**42.25** (21.3-69.1)	6	**38.4** (33.6-43.6)	6	**38.9** (28.5-67.1)
		10^9^cells/L	8	**0.04** (0.01-0.13)	6	**0.01** (0.004-0.04)	9	**0.02** (0.004-0.06)	14	**0.01** (0.001-0.07)	6	**0.01** (0.002-0.02)	6	**0.005** (0.0006-0.02)
CD3+CD4+CD25hiCD127low CCR4+CD45RO+ (Effector Treg)	CD4+ CD25hi CD127low	%	8	**28.85** (14.5-35.8)	6	**28.5** (22.2-43)	9	**35** (22.6-43.2)	14	**42.85** (21.5-71)	6	**50.3** (46.3-51.7)	6	**48.6** (23.6-58.3)
		10^9^cells/L	8	**0.02** (0.004-0.06)	6	**0.01** (0.002-0.03)	9	**0.01** (0.002-0.04)	14	**0.01** (0.001-0.07)	6	**0.01** (0.003-0.02)	6	**0.006** (0.0005-0.02)
CD3+CD4+CD25hiCD127low CCR4+CD45RO+HLA-DR+ (Activated effector Treg)	Effector Treg	%	8	**22.8** (16.2-39.3)	6	**32.6** (16.5-41)	9	**34.4** (24.9-51)	14	**25.15** (12.9-36.2)	6	**22.45** (14.6-34)	6	**16.95** (10-30)
		10^9^cells/L	8	**0.014** (0.004-0.065)	6	**0.01** (0.002-0.025)	9	**0.012** (0.002-0.04)	14	**0.006** (0.001-0.04)	6	**0.003** (0.001-0.01)	6	**0.002** (0.0002-0.01)

The values in bold are the "median" and the values in brackets are the min-max.

**Table:** 

B) Breg and memory Breg values														
Breg/subset	From	Cell frequency	1-3 years		> 3-5 years		> 5-7 years		> 7-10 years		> 10-14 years		>14-18 years	
			(n)	Median (min-max)	(n)	Median (min-max)	(n)	Median (min-max)	(n)	Median (min-max)	(n)	Median (min-max)	(n)	Median (min-max)
CD19+CD24hiCD38hi (Transitional Breg)	CD19+ (B cell)	%	12	**13.75** (6.4-26.9)	6	**6.58** (4.18-22.1)	9	**8.04** (4.58-10.4)	14	**6.82** (2.57-12.7)	6	**7.9** (3.46-10.3)	5	**10.3** (5.63-12.8)
		10^9^cells/L	12	**0.12** (0.03-0.5)	6	**0.03** (0.01-0.14)	9	**0.04** (0.01-0.07)	14	**0.03** (0.003-0.15)	6	**0.02** (0.01-0.04)	5	**0.02** (0.004-0.04)
CD19+CD24hiCD27+ (Memory Breg)	CD19+ (B cell)	%	8	**5.28** (2.61-9.52)	6	**9.1** (5.6-11)	9	**11** (4.79-21.9)	14	**10.83** (1.73-19.8)	6	**8.76** (4.62-22.4)	5	**9.88** (4.65-19.9)
		10^9^cells/L	8	**0.042** (0.01-0.17)	6	**0.04** (0.01-0.1)	9	**0.06** (0.01-0.15)	14	**0.042** (0.002-0.2)	6	**0.02** (0.01-0.08)	5	**0.02** (0.003-0.06)

The values in bold are the "median" and the values in brackets are the min-max.

### 3.3 Treg undergo a maturation process with age

Regarding the maturation process of Treg, the maturation status increased with age. Specifically, naïve Treg (CD4^+^CD25^hi^CD127^low^CCR4^-^CD45RO^-^) presented a strong negative correlation with age (r = -0.600 p = 5.1E-6), and this decrease was significantly prominent from the age of 7 onwards compared with the youngest groups (median 58% at 5-7 years vs 42.25% at 7-10 years; p = 0.023) (**Figure 2E**; **Supplementary Figure 4E**). In contrast, the eTreg cells (CD4^+^CD25^hi^CD127^low^CD45RO^+^CCR4^+^) presented a strong positive correlation with age (r = 0.660; p = 2.55E-7) (**Figure 2F**; **Supplementary Figure 4F**). The increase in both subsets was prominent from the age of 7 onwards, which correlates with a parallel decrease in naïve cells. Interestingly, activated eTreg (CD4^+^CD25^hi^CD127^low^ CD45RO^+^CCR4^+^HLA-DR^+^) showed an increasing trend from 3 to 7 years, were more marked in the 5–7-year range (median 22.8% at 1-3 years vs 34.4% at 5-7 years; p = 0.012), and the frequency decreased thereafter (**Supplementary Figure 4G**). In summary, mature Treg populations increase from the age of 7, while the naïve population decreases. The age range interval from 3 to 7 years seems to be important for the maturation process of Treg.

### 3.4 Breg are an abundant population during the first years of life

**Figure 3:**
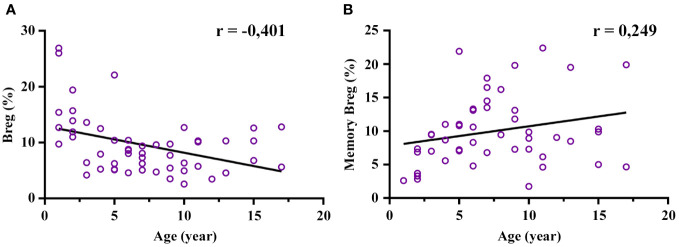
Correlation of regulatory B cell (Breg) frequency with age. The total Breg (CD19^+^CD24^hi^CD38^hi^) frequency decreases mainly after 3 years of age along with an increase in memory Breg (CD19^+^CD24^hi^CD27^+^). **(A)** Moderate negative correlation of Breg frequency (%) with age (statistically significant). **(B)** Low positive correlation of memory Breg with age (tendency: it reached no statistical significance). The frequency of both Breg and memory Breg was calculated from CD19^+^. Low association was between 0.1-0.3; moderate positive association was between 0.3-0.5; and strong positive association was between 0.5-1.

The age-stratified transitional Breg (CD19^+^CD24^hi^CD38^hi^) and memory Breg (CD19^+^CD24^hi^CD27^+^) relative frequencies and absolute numbers are shown in **Table 1B**. The frequency of transitional Breg showed a moderate negative correlation with age (r = -0.401; p = 0.003), and this decrease was clearly prominent from 3 years of age onwards (**Figure 3A**; **Supplementary Figure 5A**). In fact, the median of transitional Breg dropped from 13.75% at 1-3 years to 6.58% at 3-5 years (p = 0.049). In contrast, memory Breg (CD19^+^CD24^hi^CD27^+^) presented a low positive correlation with age although it did not reach statistical significance since this increase was mainly prominent from the age of 3 years onwards (5.28% at 1-3 years to 9.055% at 3-5 years; p = 0.039) and remained stable thereafter (**Figure 3B**; **Supplementary Figure 5B**). In summary, transitional Breg has an abundant population during the first years of life and undergoes a maturation process early on in the first 3 years of life.

## 4 Discussion

Treg and Breg are of increasing interest in the field of IEI as a breakdown of immune homeostasis may result in both autoimmunity and immunodeficiency (13, 14, 54). Although most PIRDs are early-onset diseases (2, 3, 13, 14), in the present literature, there are limited well-established reference values for the regulatory population in the pediatric population. Herein, we describe the changes observed in both Treg and Breg in a healthy pediatric population (1-18 years). Our main findings suggest: 1) Treg and Breg are abundant populations before the age of 7 and 3, respectively, presenting special biological meaning as the immunotolerance process occurs during early childhood, and 2) the three different definitions of Treg (see below) are highly comparable between them. Therefore, biologically, our preliminary data contribute to improving the understanding of the normal biological course of regulatory populations during early life, which could be of interest in subsequent studies with larger cohorts. Clinically, our study is of interest for the clinical diagnosis of IEI including PIRDs.

A consensus on Treg phenotypical definition is needed for a better characterization of Treg for both clinical and research use (39). The most accurate phenotypic definition for Treg is CD4^+^CD25^hi^CD127^low^FoxP3^+^ (39) since FoxP3 is essential for Treg immune suppressive activity (65), and the inclusion of FoxP3 reduces the variability in the % of Treg (39). Also, the marker CD127 is key to discerning between CD127^+^ T cells from Treg (CD127^low^) (66, 67). Herein, we studied the correlation between the different definitions described in the current literature to verify their equitability. Overall, the preliminary results suggested a strong correlation between 1) the extracellular definitions: CD4^+^CD25^hi^ vs CD4^+^CD25^hi^CD127^low,^ and 2) the two intracellular definitions: CD4^+^CD25^hi^CD127^low^FoxP3^+^ vs CD4^+^CD25^hi^FoxP3^+^. The correlation between the extracellular and intracellular definitions was moderately positive for most age ranges (1-18 years), so we cannot confirm their full interchangeability with the current results. However, the correlation between CD4^+^CD25^+^CD127^low^ and CD4^+^CD25^+^Foxp3^+^ cells was previously confirmed with a strong correlation in a study including 43 healthy donors above 18 years of age (66). This indicates that the findings observed in adults cannot be fully extrapolated to pediatric populations and highlights the need for reference values by age range, including those of regulatory cells.

In addition, depending on the Treg definition, the frequency of Treg is in different interval scales, making it difficult to interchange results for clinical interpretation. For instance, our results showed that the relative frequency in a healthy pediatric population (1-18 years) for CD4^+^CD25^hi^CD127^low^ oscillates between 3.3–9.7% of CD4^+^ cells, which was consistent with another study showing 2.2-7.7% of CD4^+^ cells (1-18 years; n = 81) (35). However, when we analyzed Treg with the definition CD4^+^CD25^hi^CD127^low^FoxP3^+^, it fell to 0.07-1.6% of CD4^+^ cells. The current Working Definitions for Clinical Diagnosis of Primary Immunodeficiency recommend studying FoxP3 expression in CD4^+^CD25^hi^ cells (21). However, at present, there is no consensus on the definition of Treg nor are there reference values in pediatric populations for all three definitions (36). Because of this inter-laboratory variation, and despite the correlation between values from different Treg cell definitions, we stress the need for age-range reference values of the regulatory populations specific for all three definitions.

Overall, Treg decreases with age; this decrease is more evident when the CD127 marker is included (CD4^+^CD25^hi^CD127^low^ and CD4^+^CD25^hi^CD127^low^FoxP3^+^) and is especially prominent after the age of 7 years. This result was consistent with a previous study reporting a slight decline in Treg (CD4^+^CD25^hi^CD127^low^) frequency with age (35). Regarding the maturation process of Treg, we saw an increment of memory Treg (CD4^+^CD25^hi^CD127^low^CD45RO^+^) with age (strong correlation; data not shown). Similarly, R. van Gent et al. and M. Garcia-Prat et al. described an evident increase in memory Treg (CD4^+^CD25^hi^CD127^low^CD45RO^+^), with the same markers we used (35, 36). This increase showed significant differences with the groups older than 7 years (36). Regarding this last point, we observed an increase in eTreg (CD4^+^CD25^hi^CD127^low^CD45RO^+^CCR4^+^) and a decline of naïve Treg CD4^+^CD25^hi^CD127^low^CD45RO^-^CCR4^-^), mainly from the age of 7 years. In addition, we studied the activated eTreg (CD4^+^CD25^hi^CD127^low^CD45RO^+^CCR4^+^HLA-DR^+^), which presented a special increment from 5-7 years of age and then underwent a marked decrease. The age range interval from 3 to 7 years thus seems to be important for the maturation process of Treg. This increase in activated eTreg could be explained by the need to generate tolerance for the transition from perinatal life to the first encounters in infancy with new environmental antigens (dietary changes, aeroallergens, and nursery), especially for harmless antigens (68–71). In summary, although our results are preliminary, they suggest that the Treg profile undergoes a change in its maturational profile at around 7 years of age, with a decline of naïve Treg and the increment of memory/effector Treg.

Transitional Breg (CD19^+^CD24^hi^CD38^hi^) is an abundant population during the perinatal period (pre- and post-natal), promoting tolerogenic responses during pregnancy and at birth. In fact, we (50) and Sarvaria et al. (63) demonstrated that transitional Breg is a highly frequent population in human umbilical cord blood with potent inhibitory activity such as the suppression of Th1 cell differentiation and effector functions while enhancing Treg activity (47–49). For instance, transitional Breg from cord blood could be of benefit to mitigate chronic graft-versus-host disease after hematopoietic transplantation when using this source as opposed to bone marrow since these IL-10 B cells present a strong inhibitory capacity (63). After birth, transitional Breg is known to be important for peripheral immuno-tolerance and their dysregulation has been associated with autoimmune conditions, such as juvenile dermatomyositis (72) and arthritis (52), and more recently, immunodeficiency including CVID (53). The results of the present study further reinforce the notion that Breg remains an abundant population up to 3 years of age along with a progressive increment of memory Breg with age.

Currently, CD19^+^CD24^hi^CD38^hi^ is the most widely accepted phenotypical definition for Breg (48, 50, 56–59). However, as the term Breg is a collective one for those B cells that exhibit immunosuppressive capacity, all B cells may present the capacity to differentiate into IL-10-producing cells depending on the environmental requirement although some subsets are more suitable than others (73). Thus, B cells from different developmental stages can exercise regulatory functions, such as immature B cells (CD19^+^CD24^hi^CD38^hi^) (48, 50, 56–59), mature B cells (CD19^+^CD24^hi^CD27^+^) (60), and plasmablast cells (CD19^+^CD138^hi^TACI^+^CXCR4^+^CD1^dint^Tim1^int^Blimp-1^+^IgG^-^) (73). Here, we evaluated the changes of CD19^+^CD24^hi^CD38^hi^ cells in a pediatric population, accepting the limitation that CD19^+^CD24^hi^CD38^hi^ cells resemble the transitional B cell phenotype (CD19^+^IgM^+^CD38^hi^) (36, 62). In fact, our results are consistent with those described by M. Garcia-Prat et al. showing a decline of transitional B cell (CD19^+^CD24^hi^CD38^hi^) frequency with age, and this decrease was prominent after the age range of 3-4 years (36).

As the number of PIRD cases increases, the need to better characterize Treg and Breg to better understand the pathophysiology of these diseases becomes greater. At present, there is still a discrepancy in the denotation of Treg and Breg, both in their phenotypic and functional characterization and much less is known about their subpopulations. In this context, our work differs from previous studies by analyzing the most common definitions of Treg in a healthy pediatric population since the gating strategy remains a non-consensus issue (18, 35, 37, 38, 40–43). Recognizing the limitation of sample size, our results were mostly consistent with those in the current literature. Interestingly, there is evidence that in cord blood, the frequency and functionality of female Treg is higher than in males (74), suggesting that gender is an important factor to consider when analyzing regulatory subsets. However, we did not include this variable due to the small sample size. Furthermore, when establishing normality parameters for general populations, it is crucial to take ethnicity into account. Nevertheless, all the healthy controls in this study are of Caucasian origin. Hence, we encourage a comprehensive and large sample size analysis of regulatory cells in future work to establish age-stratified reference values in healthy pediatric populations including these two variables.

Herein, we analyzed CD25 and FoxP3 expression. However, other functional markers exist to define Treg (i.e., CTLA-4, ICOS, and PD-1), and most of them are assessable by flow cytometry. CTLA-4 is a co-inhibitory surface molecule that is constitutively expressed in Treg and its expression assay is of great interest for the diagnosis and clinical management of both PIRDs (75–78) and autoimmune diseases (rheumatic diseases) (79). For instance, we and others analyzed the CTLA-4 expression in Treg to evaluate patients with immune dysregulation when no genetics were identified (75–77). In addition, given the suppressive capacity of CTLA-4, the use of CTLA-4-Ig (fusion protein: IgG1 Fc+CTLA-4) is an effective approach in treating CTLA-4 haploinsufficiency. Indeed, CTLA-4 expression in Treg has recently been used in the field of rheumatology for the evaluation of abatacept (fusion protein: IgG1 Fc+CTLA-4) responses (79). Mainly, the use of abatacept resulted in the reduction of IL-6 (inflammatory cytokines) and the normalization of Treg frequency after 6-12 months of treatment (79). However, in the field of PIRD, CTLA-4 expression in Treg has not been included in the ESID diagnostic criteria as a standardized tool for diagnosis (13). We believe it could be of much interest. Besides Treg’s phenotypic description, functional assays are of paramount importance for the diagnosis of PIRDs as deficiency of suppressor capacity needs to be correlated to an altered Treg frequency. Current Treg cell suppression assays are based on the *in vitro* co-culture of Treg cells and T cells and the evaluation of the T cell proliferation rate (80). This functional assay has limitations, such as 1) the difficulty of extrapolating the results to *in vivo* conditions, 2) the inability to evaluate each inhibitory mechanism separately since suppression of T-cell proliferation is the result of Treg inhibitory mechanisms as a whole, and 3) the technical implementation in the clinical setting due to sample availability, processing time, costs, and technical complexity. In future studies, we suggest designing new strategies for Treg functional assay implementation.

To conclude, there is a good level of correlation between the Treg definitions (CD4^+^CD25^hi^CD127^low^, CD4^+^CD25^hi^CD127^low^FoxP3^+^, and CD4^+^CD25^hi^FoxP3) and CD4^+^CD25^hi^, mainly when comparing the two intracellular panels (CD4^+^CD25^hi^CD127^low^FoxP3^+^ vs CD4^+^CD25^hi^FoxP3^+^) and the two extracellular panels (CD4^+^CD25^hi^ vs CD4^+^CD25^hi^CD127^low^). The numerical values of Treg frequency span a wide range in all ages depending on the marker combinations; the median oscillates between 0.64 – 7.31% of CD4^+^ cells. Our results suggest that the total frequency of both Treg and Breg tend to decline after 7 and 3 years onwards, respectively, along with a maturation process with age. Based on these results, a consensus on which Treg definition to use and age-stratified reference values for regulatory populations for each definition are needed for the clinical diagnosis of IEI.

## Data availability statement

The original contributions presented in the study are included in the article/**Supplementary Material**. Further inquiries can be directed to the corresponding authors.

## Ethics statement

The studies involving humans were approved by the Ethics Committee of the Hospital Sant Joan de Déu (Comité Ético de Investigaciones Clínicas number PIC-129-18). The studies were conducted in accordance with the local legislation and institutional requirements. Written informed consent for participation in this study was provided by the participants’ legal guardians/next of kin.

## Author contributions

YL: Conceptualization, Data curation, Formal Analysis, Investigation, Methodology, Project administration, Resources, Software, Validation, Visualization, Writing – original draft, Writing – review & editing. DA: Writing - original draft, Formal analysis, Methodology, Visualization. AV: Data curation, Validation, Writing – review & editing. AC: Methodology, Writing – review & editing. AG-G: Methodology, Writing – review & editing. AD-M: Data curation, Validation, Writing – review & editing. CM-C: Methodology, Writing – review & editing. AE-S: Conceptualization, Data curation, Investigation, Supervision, Validation, Writing – original draft, Writing – review & editing. LA: Conceptualization, Data curation, Funding acquisition, Investigation, Project administration, Supervision, Validation, Writing – original draft, Writing – review & editing.
