# Supplementary material for: Changes in Treg and Breg cells in a healthy pediatric population
Source: Front Immunol. 2023 Nov 21;14:1283981. doi: 10.3389/fimmu.2023.1283981 (PMC10704817; doi:10.3389/fimmu.2023.1283981)

Supplementary Material

Supplementary Figure 1. Regulatory T cell (Treg) immunophenotyping gating strategy. A) Extracellular panel: $CD4^+CD25^{hi}CD127^{low}$, naïve Treg ($CD4^+CD25^{hi}CD127^{low}CD45RO^-CCR4^-$), memory Treg ($CD4^+CD25^{hi}CD127^{low}CD45RO^+CCR4^+$) and activated effector Treg ($CD4^+CD25^{hi}CD127^{low}CD45RO^+CCR4^+HLA\text{-}DR^+$). B) Intracellular panels: $CD4^+CD25^{hi}CD127^{low}Foxp3^+$, and $CD4^+CD25^{hi}FoxP3^+$.

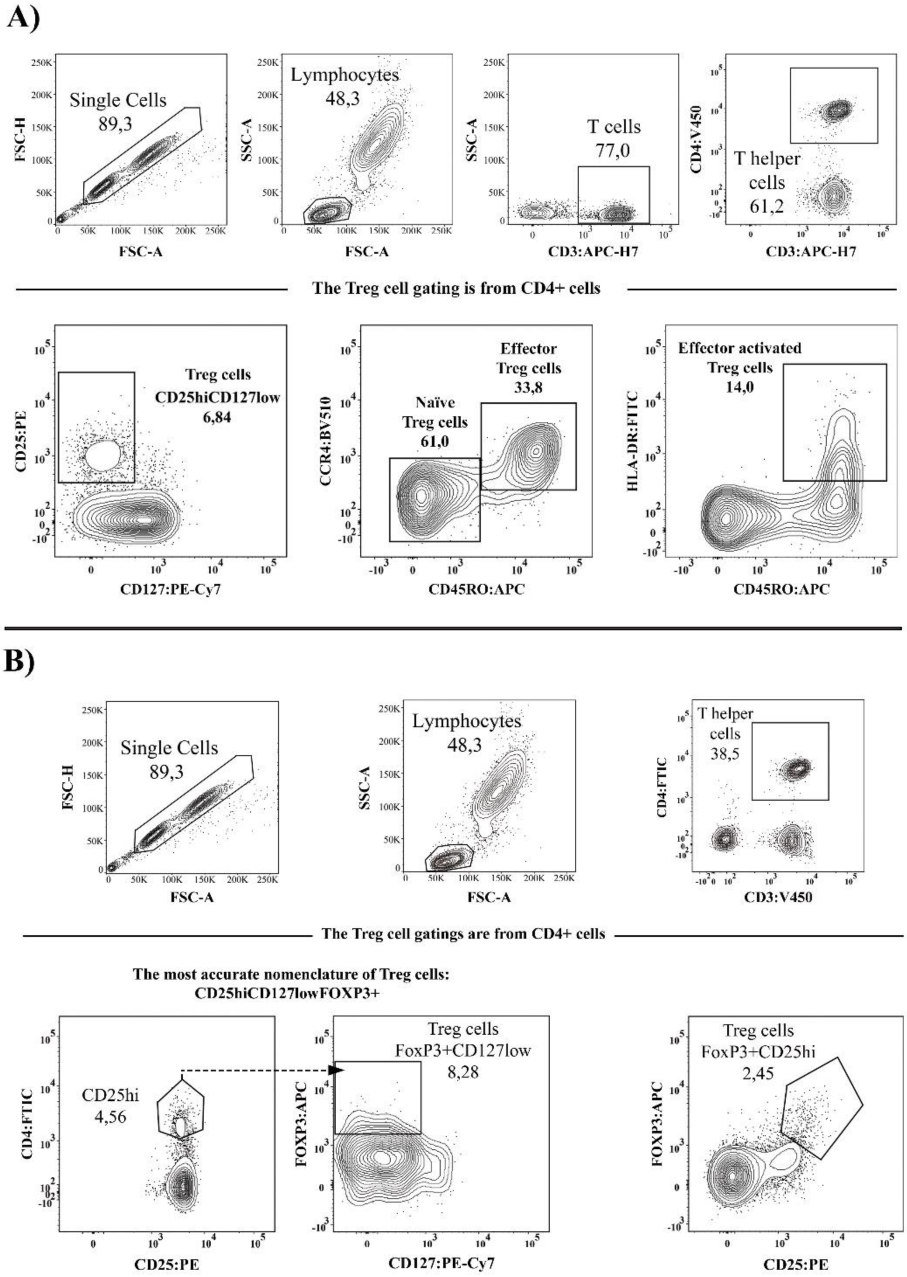

Supplementary Figure 2. Regulatory B cell (Breg) immunophenotyping gating strategy. Transitional Breg is defined as $CD19^{+}CD24^{hi}CD38^{hi}$ and memory Breg cell as $CD19^{+}CD24^{hi}CD27^{+}$.

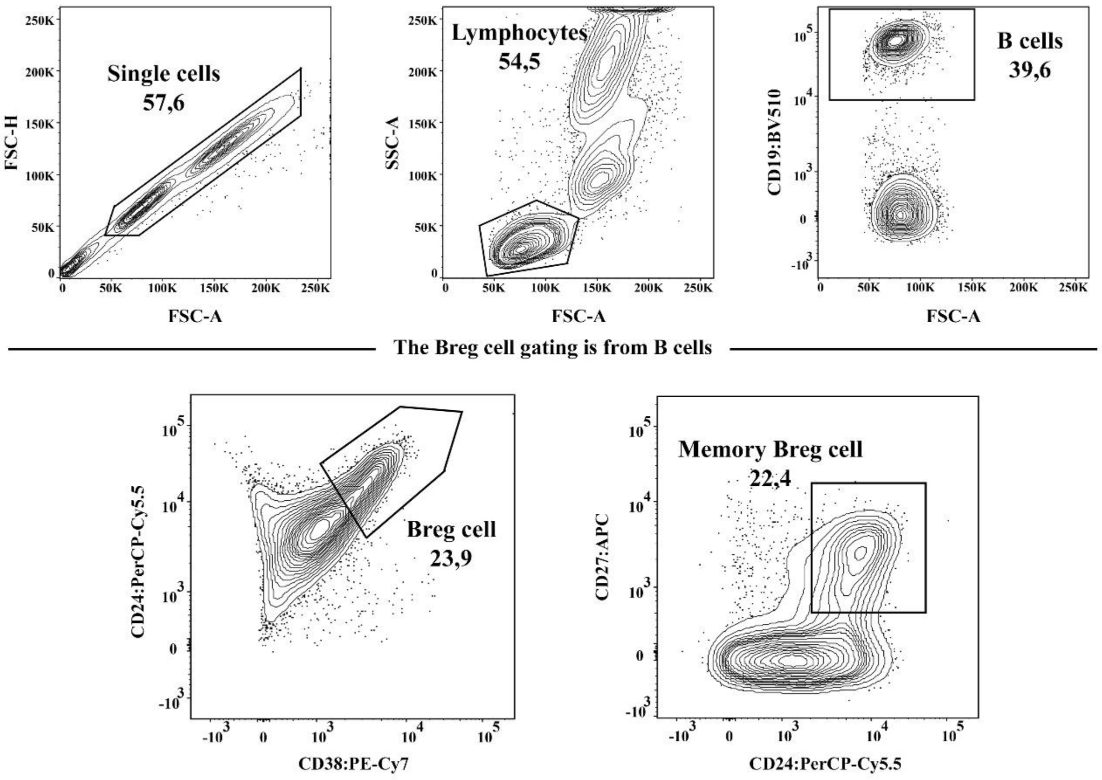

Supplementary Figure 3. Correlation between gating strategies of regulatory T cell (Treg) and $CD4^{+}CD25^{hi}$ by age. The three Treg definitions are $CD4^{+}CD25^{hi}CD127^{low,}$ $CD4^{+}CD25^{hi}CD127^{low}Foxp3^{+}$, and $CD4^{+}CD25^{hi}FoxP3^{+}$. The correlation between the three Treg definitions was moderate-strong for most of age ranges, being especially strong between the intracellular panels, and $CD4^{+}CD25^{hi}$ vs $CD4^{+}CD25^{hi}CD127^{low}$. The values inside the boxes indicate the Spearman correlation strength. Low association 0.1-0.3; moderate positive association between 0.3-0.5; and strong positive association 0.5-1. (*) p value ≤ 0.05; (**) p value ≤ 0.01.

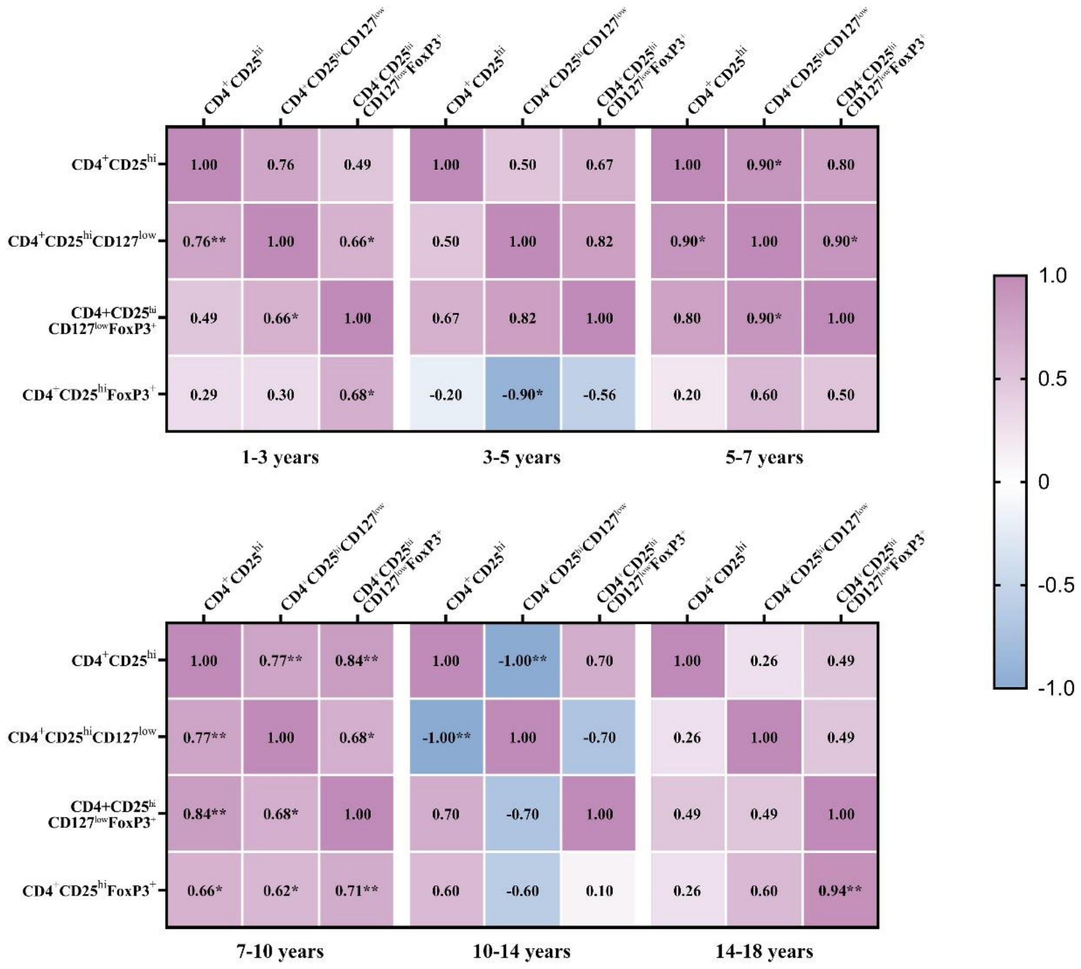

Supplementary Figure 4. Frequency of regulatory T cell (Treg) by age range. A), **B)**, **C)** and **D)** Total Treg (median/%) decreases mainly after 7 years onwards. **E)** Decrease of naïve Treg (CD4$^+$CD25^{hi}CD127^{low}CD45RO$^-$CCR4$^-$) with age. **F)** Increase of effector Treg (CD4$^+$CD25^{hi}CD127^{low}CD45RO$^+$CCR4$^+$) with age. **G)** Increment of effector activated Treg (CD4$^+$CD25^{hi}CD127^{low}CD45RO$^+$CCR4$^+$HLA-DR$^+$) between 3-7 years. **E), F)** and **G)** suggest a process of maturation of Treg with age. The frequency of CD25, CD25^{hi}CD127^{low}, CD25^{hi}CD127^{low}FoxP3$^+$ and CD25^{hi}FoxP3$^+$ are calculated from the CD4$^+$ cells. The naïve and effector Treg are from CD25^{hi}CD127^{low}. (*) p value $\leqslant$ 0.05; (**) p value $\leqslant$ 0.01. Discontinuous line: quartiles; Black solid line: median.

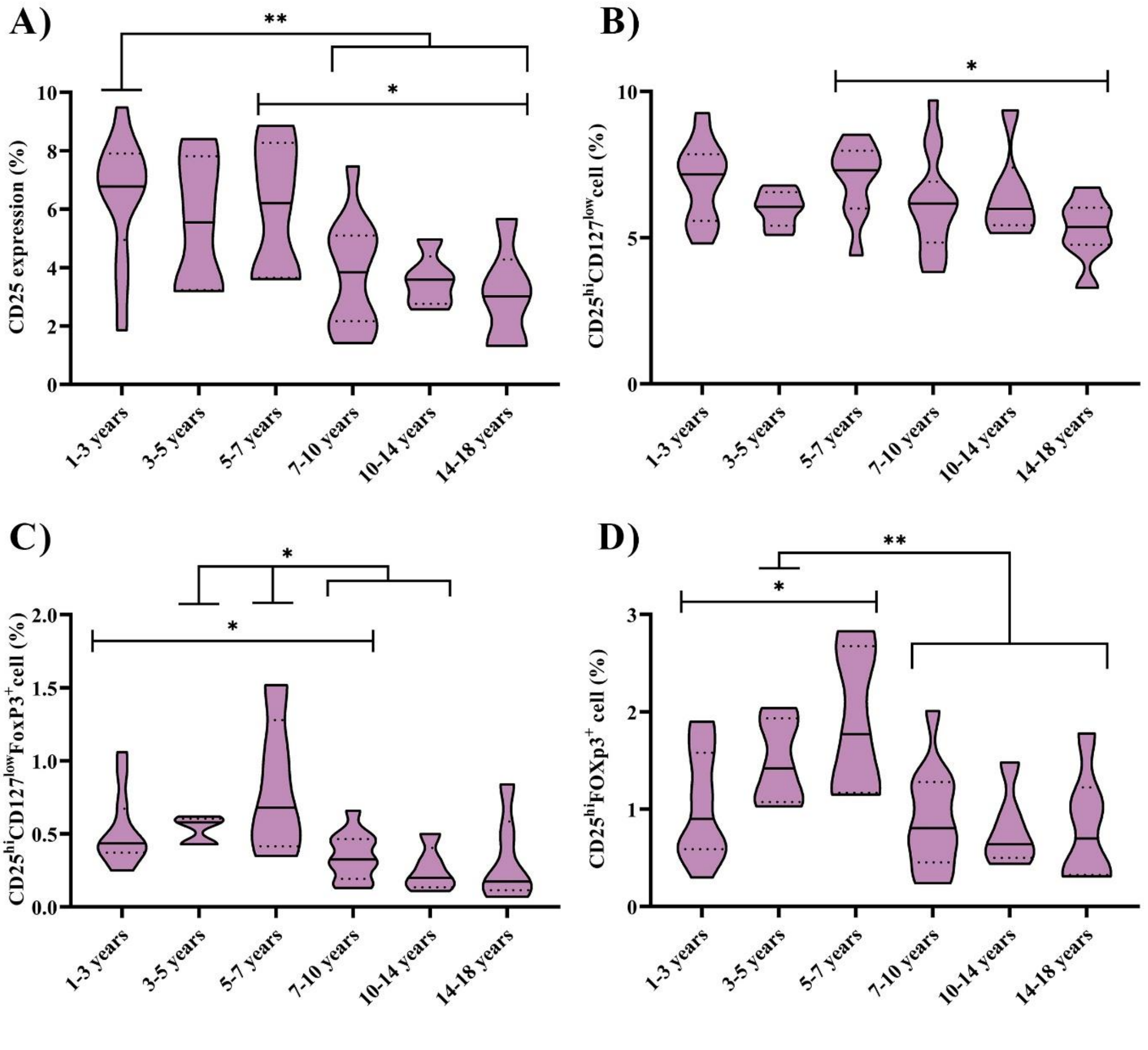

(continued on next page)

(continued)

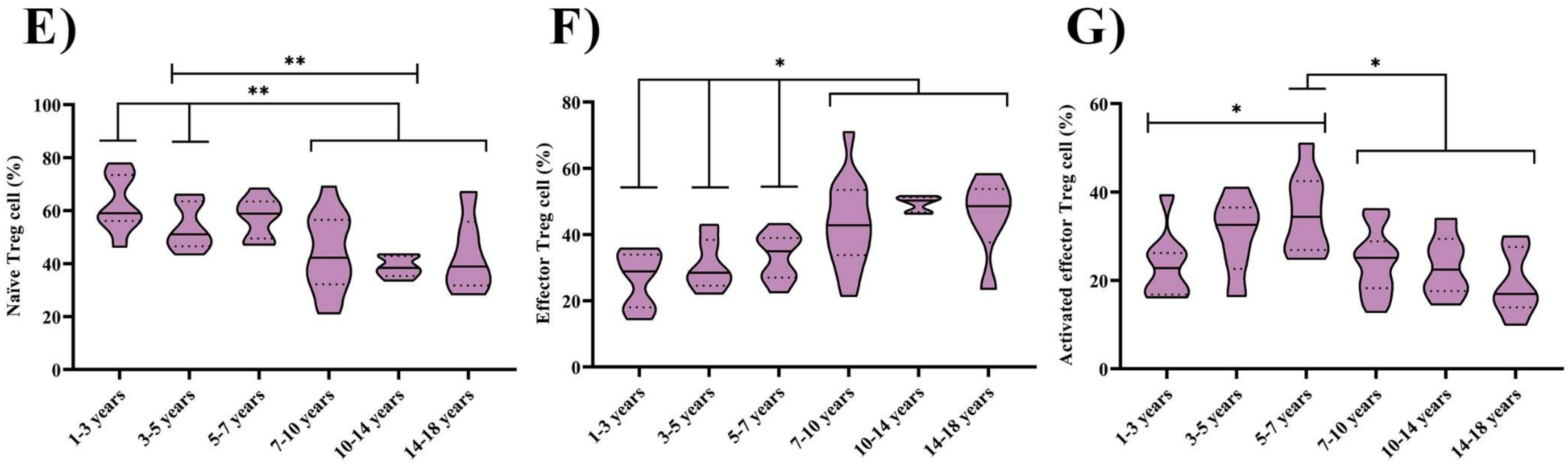

E)
**
**
100
80
60
40
20
0
Naïve Treg cell (%)
1-3 years
3-5 years
5-7 years
7-10 years
10-14 years
14-18 years
F)
*
80
60
40
20
0
Effector Treg cell (%)
1-3 years
3-5 years
5-7 years
7-10 years
10-14 years
14-18 years
G)
*
*
60
40
20
0
Activated effector Treg cell (%)
1-3 years
3-5 years
5-7 years
7-10 years
10-14 years
14-18 years

Supplementary Figure 5. Frequency of regulatory B cell (Breg) by age range. A) The total Breg (CD19^{+}CD24^{hi}CD38^{hi}) (median/%) decrease mainly after 3 years onwards. **B)** Increment of memory Breg (CD19^{+}CD24^{hi}CD27^{+}) frequency with age. The frequency of both Breg and memory Breg was calculated from CD19^{+}. (*) p value $\leqslant 0.05$; (**) p value $\leqslant 0.01$. Discontinuous line: quartiles; Black solid line: median.

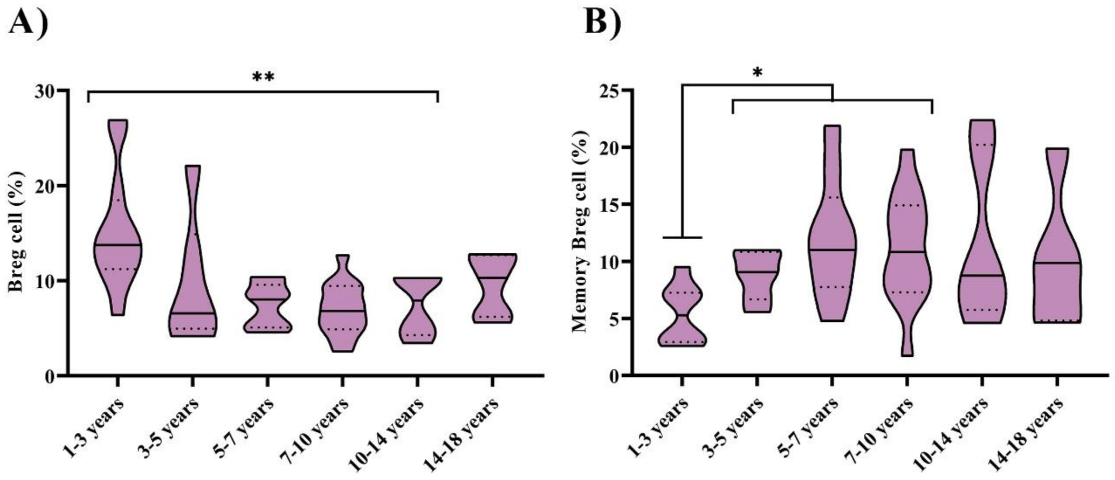

Supplement: Supplementary file 1 [file DataSheet_1.pdf]
